# TargetCLP: clathrin proteins prediction combining transformed and evolutionary scale modeling-based multi-view features via weighted feature integration approach

**DOI:** 10.1093/bib/bbaf026

**Published:** 2025-01-23

**Authors:** Matee Ullah, Shahid Akbar, Ali Raza, Kashif Ahmad Khan, Quan Zou

**Affiliations:** Institute of Fundamental and Frontier Sciences, University of Electronic Science and Technology of China, Chengdu, Sichuan 610054, China; Institute of Fundamental and Frontier Sciences, University of Electronic Science and Technology of China, Chengdu, Sichuan 610054, China; Department of Computer Science, Abdul Wali Khan University Mardan, Mardan 23200, Pakistan; Department of Computer Science, MY University, Islamabad 45750, Pakistan; Department of Computer Science, Abdul Wali Khan University Mardan, Mardan 23200, Pakistan; Institute of Fundamental and Frontier Sciences, University of Electronic Science and Technology of China, Chengdu, Sichuan 610054, China; Yangtze Delta Region Institute (Quzhou), University of Electronic Science and Technology of China, Quzhou, Zhejiang 324003, China

**Keywords:** clathrin protein, sequence analysis, feature integration, protein language model, transformed features, self-normalized model

## Abstract

Clathrin proteins, key elements of the vesicle coat, play a crucial role in various cellular processes, including neural function, signal transduction, and endocytosis. Disruptions in clathrin protein functions have been associated with a wide range of diseases, such as Alzheimer’s, neurodegeneration, viral infection, and cancer. Therefore, correctly identifying clathrin protein functions is critical to unravel the mechanism of these fatal diseases and designing drug targets. This paper presents a novel computational method, named TargetCLP, to precisely identify clathrin proteins. TargetCLP leverages four single-view feature representation methods, including two transformed feature sets (PSSM-CLBP and RECM-CLBP), one qualitative characteristics feature, and one deep-learned-based embedding using ESM. The single-view features are integrated based on their weights using differential evolution, and the BTG feature selection algorithm is utilized to generate a more optimal and reduced subset. The model is trained using various classifiers, among which the proposed SnBiLSTM achieved remarkable performance. Experimental and comparative results on both training and independent datasets show that the proposed TargetCLP offers significant improvements in terms of both prediction accuracy and generalization to unseen data, furthering advancements in the research field.

## Introduction

Clathrin proteins are the well-known adaptor proteins that work as the basic components of the vesicle-coating complex and are useful for the membrane cleavage to release the invaginated vesicle from the plasma membrane [1]. It involves a heavy chain having a molecular weight of ‘180 kDa’ with two light chains of ‘α’ and ‘β’ to form a tri-legged protein [2]. It has been reported that clathrin-mediated endocytosis is a fundamental stage in cell regulation [3]. The plasma membrane proteins and vesicles are interconnected using clathrin and are moved from the plasma membrane to the intercellular for localizing and prediction concerns. Clathrin also effectively performs cell division [4]. According to several studies, the absence of clathrins in the human body may cause several killer diseases, including Alzheimer’s, cancer, and neurodegenerative disorders [5, 6].

Identifying the substantial role of clathrins in human diseases has attracted scientists to further explore this research area. Many biological methods such as agarose gel electrophoresis [7], partial amino acid Sequence [8], Tom1–Tollip complex [9], and different molecules-based proteolysis [10] to predict the clathrin-binding domain have shown remarkable progress in predicting clathrins. In the last decade, computational analysis-based protein modeling has provided an alternative method to correctly identify several biological proteins rapidly with low operational cost [11, 12].

In the literature, few computational predictors have been presented to predict clathrin sequences [13]. Khanh Le et al. proposed a 2D convolutional neural network (CNN) to predict clathrin proteins [4]. The evolutionary descriptors were collected from the protein sequences by representing each sequence in the form of a 2D image. However, this model considers only evolutionary features without keeping the physiochemical properties-based structure information of the protein samples. Similarly, Ju Zhang et al. presented a bidirectional long short-time neural network (Bi-LSTM) and CNN-based ensemble deep learning model to predict clathrin proteins [2]. The physiochemical properties based on amino acids cluster information were extracted from each sequence. To handle unbalanced data, the random oversampling and under-sampling approach was employed to choose the optimal procedure for the training dataset. Recently, Khalid et al. proposed a machine learning-based ensemble model called CL-Pred for the prediction of clathrin proteins [14]. The clathrin sequences were numerically formulated using sequential, position-specific scoring matrix (PSSM)-based evolutionary descriptors and transformed evolutionary descriptors. The extracted features were trained using a light eXtreme gradient boosting-based ensemble training model. The proposed CL-Pred performed better than the existing methods.

Although there are few computational predictors available, there remains a need for efficient computational predictor that can further boost the predictive efficacy of clathrin proteins. Therefore, in this study, we attempt to develop a novel computational predictor to predict clathrin proteins from non-clathrin proteins. The following four key aspects are considered to enhance the performance: (i) Feature extraction: we proposed two new feature extraction methods, named PSSM-CLBP and RECM-CLBP, along with the qualitative characteristics features (QLC) and well-known ESM embeddings; (ii) Feature integration: A weighted-based feature integration approach using differential evolution (DE) was employed to integrate all the single-view features into a weighted-based multi-view representation; (iii) Classification algorithm: A new classifier, the self-normalized BiLSTM (SnBiLSTM), was specifically designed for the clathrin protein prediction task; and (iv) Generalization: the model was tested against unseen data to measure its generalization strength. Based on the proposed pipeline, we were able to implement a more powerful prediction algorithm, called TargetCLP, for clathrin proteins. Experimental results further validate the performance of TargetCLP. The data and source code used in this study are available at https://github.com/MateeullahKhan/TargetCLP.

## Datasets and methods

### Dataset

Establishing reliable, stringent, and comprehensive datasets is the foremost vital step for developing an intelligent and robust predictor. In order to fairly compare and judge the prediction performance with the past work, we derived the datasets of the Le et al., work [4]. The dataset is divided into clathrin proteins as positive samples (positive class) and non-clathrin or general proteins, with vesicular transport proteins collected as negative samples (negative class) due to their similar structure and function to positive samples. The dataset is derived from the National Center for Biotechnology Information (NCBI) [15] with protein sequences specifically chosen from the UniProt [16] data source available through NCBI.

To maximize the model effectiveness, redundant sequences with 100% similarity were removed using a common clustering biological sequence tool BLAST [17]. After the cut-off level, the non-redundant dataset contained 1546 positive and 1360 negative samples. This non-redundant dataset is then divided into benchmark training and independent datasets. The benchmark training dataset includes a total of 2421 samples, with 1288 as clathrin or positive samples and 1133 as non-clathrin or negative samples. Similarly, the independent dataset contains 485 sequences, of which 258 are clathrin and 227 are non-clathrin proteins. In our study, we represent the training and independent datasets as CL2421 and CL485, respectively. Additionally, to understand the biological significance of the proteins, the datasets were also analyzed for subcellular localization using DeepLoc 2.0 [18]. The statistical summary is provided in Tables S1, S2 and Figures S1 and S2 under Text S1 in SI.

### Feature representation schemes

One of the most crucial and challenging tasks in computational biology is how to represent a biological sequence using a discrete model or vector while retaining significant sequence-order information and key pattern characteristics [19–24]. This is because the majority of the machine-learning algorithms can only process vectors [25, 26]. However, a straightforward feature representation approach, such as amino acid composition (AAC) [27, 28], defined within a discrete model may fail to preserve any sequence pattern information. Various feature extraction methods are proposed to prevent this loss of sequence-pattern information [29–31]. In addition, the use of deep-learned features is shown to be effective for efficiently developing a computational model [32–38].

In this study, along with the deep-learned embedding, we introduce new image-based feature representation techniques to convert each protein sequence into a numerical vector. The specifics of these extraction methods are outlined below.

### Representation of protein sequence as a PSSM

The PSSM, utilized to represent the evolutionary information in a provided protein sequence, has demonstrated remarkable efficacy in a range of bioinformatics-based prediction tasks and, hence, found widespread application including deciphering protein folding mechanisms [39], identifying protein-ligand binding sites [40], protein contact map prediction [41] analyzing protein secondary structures [41] etc.

However, when designing machine learning-based predictors for clathrin proteins, for instance, TargetCLP in this work, PSSM, with a length equal to the provided protein sequence, cannot be employed directly to represent the clathrin protein’s feature vector. Therefore, in this study, we employed an image-based feature representation technique completed local binary pattern (CLBP) to transform the PSSMs into feature vectors of equal size.

### PSSM transformation to feature vector using CLBP

The CLBP, as proposed in [42], improves the capabilities of the local binary pattern by reflecting additional information in the image neighborhood locally. CLBP computes three elements to characterize the local region: the central pixel, the difference in magnitudes, and the difference in sign. The CLBP_Center operator, or CLBP_C, specifically focuses on the central pixel. The CLBP-Sign or CLBP_S operator calculates the difference in signs between the intensity values of the center pixel and its neighboring pixels. Similarly, the CLBP-Magnitude or CLBP_M operator computes the absolute difference in magnitude between the intensity values of the center pixel and its neighboring pixels.

Leveraging the strength of CLBP, we introduced a novel feature representation technique utilizing CLBP. We named this technique as CLBP-PSSM. The details of the CLBP-PSSM are outlined below:

For a given protein sequence of length $L$, its PSSM $P$ with the size of $L\times 20$, where 20 denotes the number of standard amino acids, can be constructed using the PSI-BLAST [43]. The PSSM $P$ is then transformed to a PSSM image ${P}_I$ with the pixel intensity values ranges from 0 to 255. ${P}_I(i,j)$ represent the intensity value at the position $(i,j)$ in the PSSM image ${P}_I$, where $i$ is the position along the sequence (row) and $j$ is the position of the amino acid type (column). Let ${P}_I\left(c,c\right)$ be the intensity value of the center pixel at the position $c$, the CLBP_C can then be calculated as:


(1) \begin{equation*} CLBP\_C=\left\{\begin{array}{@{}l}1,\text{if}\kern0.5em {P}_I\left(c,c\right)\ge T\\{}0,\text{if}\kern0.5em {P}_I\left(c,c\right)<T\end{array}\right. \end{equation*}

where $T$ is the average threshold value of the whole PSSM image ${P}_I$ used to binarize the intensity value ${P}_I\left(c,c\right)$. Similarly, the CLBP_S can be calculated as:


(2) \begin{equation*} CLBP\_S=\sum_{n=0}^{N-1}\left({s}_n\right){2}^n,\kern1.25em {s}_n=\left\{\begin{array}{@{}l}1,\text{if}\kern0.5em \left({P}_I\left(i,j\right)-{P}_I\left(c,c\right)\right)\ge 0\\{}-1,\text{if}\kern0.5em \left({P}_I\left(i,j\right)-{P}_I\left(c,c\right)\right)<0\end{array}\right. \end{equation*}

where $N$ represents the total number of involved neighbors, ${s}_n$ is the sign of $\left(P\left(i,j\right)-P\left(c,c\right)\right)$. The CLBP_M can be calculated as:


(3) \begin{equation*} CLBP\_M=\sum_{n=0}^{N-1}\left({m}_n\right){2}^n,\kern0.75em {m}_n=\left\{\begin{array}{@{}l}1,\text{if}\kern0.5em \left({P}_I\left(i,j\right)-{P}_I\left(c,c\right)\right)\ge{T}_m\\{}0,\text{if}\kern0.5em \left({P}_I\left(i,j\right)-{P}_I\left(c,c\right)\right)<{T}_m\end{array}\right. \end{equation*}

Where ${m}_m$ is the magnitude of $\left(P\left(i,j\right)-P\left(c,c\right)\right)$. The threshold value, denoted by ${T}_m$ is determined by computing the mean value of ${m}_m$ from the whole PSSM image ${P}_I$.

Finally, all the three operators are combined to construct the final PSSM-CLBP feature vector as:


(4) \begin{equation*} PSSM- CLBP= CLBP\_C+ CLBP\_S+ CLBP\_M \end{equation*}

Since CLBP operates locally, we used a $3\times 3$ window with a radius $R=1$ and neighboring pixels $N=8$. The final $PSSM- CLBP$ vector has a dimension of 236.

### Representation and transformation of RECM to feature vector using CLBP

A protein’s structural stability arises from a large number of interactions between its amino acid residues both within and between individual residues. The energy functions, derived from known protein structures, can be used to estimate the energy contributions of these interactions [44]. However, a key limitation of these energy functions is their dependence on a pre-determined 3D structure for computing the total energy. This hinders their application for proteins lacking a 3D structure or those proteins that are intrinsically disordered. Thomas and Dill proposed a method to estimate pairwise energy for each amino acid in a protein sequence, without requiring the known structure of proteins. However, this approach may not be accurate for calculating the energy of entire proteins or intrinsically unstructured proteins [45, 46]. Therefore, to inherently capture the influence of the amino acid interactions, we used the predicted residue-wise energy contact matrix (RECM) derived in [47]. The method involved fitting the residue pairwise energies of 785 tertiary structured proteins to the 674 primary protein sequences using the least square fitting technique. The RECM, provided in supplementary Table S3 of Text S2 in SI, is a square matrix with the dimension of $20\times 20$, where the values along the diagonal denote the self-contact energies of each standard amino acid while the off-diagonal denote the predicted contact energies between any two different standard amino acids. The following method is used to obtain the matrix representation for each protein sequence and then transform this matrix into RECM-CLBP feature vector.

For a given protein sequence with length $L$, a matrix, with the dimension of $L\times 20$, is obtained. This matrix, named as an $RECM-T$, effectively converts the sequence into a series of 20 dimensional vectors where each vector inherently holds information about a specific amino acid in the sequence. The matrix can be represented mathematically as:


(5) \begin{equation*} RECM-T={\left[\begin{array}{cccc}{p}_{1,1}& {p}_{1,2}& \cdots & {p}_{1,20}\\{}{p}_{2,1}& {p}_{2,2}& \cdots & {p}_{2,20}\\{}\vdots & \vdots & \vdots & \vdots \\{}{p}_{L,1}& {p}_{L,2}& \cdots & {p}_{L,2}\end{array}\right]}_{L\times 20} \end{equation*}

where $p(j,l)\ (l=1,2,...,20)$ is the element in the $RECM-T$ matrix, $j$ is the position of the amino acid in the sequence (row), and $l$ represent the position in 20 amino acid types (column). Next, the same process as discussed in PSSM transformation using CLBP is employed to transform the $RECM-T$ matrix into $RECM- CLBP$ feature vector. The obtained novel $RECM- CLBP$ contains a total of 236 features representing each protein sequence numerically. The working mechanism of these transformations is discussed with examples in SI Text S3.

### Qualitative characteristics features

The QLC [31] represents a global formulation approach for analyzing amino acids based on their physiochemical properties distributed across an amino acid sequence. More specifically, the QLC analyzes seven key physiochemical attributes (polarity, predicted secondary structure, polarizability, charge, normalized Van der Waals volume, hydrophobicity, and solvent accessibility) of amino acid residues to group them into three distinct categories. Supplementary Table S4 under Text S4 in SI provides the details.

QLC captures the residue-wise transition, distribution, and composition of amino acids within the protein sequence based on transition (T), distribution (D), and composition (C) indices. The index T shows the likelihood probability of transitions between two adjacent amino acid residues with differing properties. The index D analyzes the distribution of amino acid residues along the protein sequence at 25%, 50%, 75%, and 100% intervals. The index C quantifies the overall composition of a sequence by computing the percentage composition of each group of amino acid residues categorized based on physiochemical properties [48]. For a given protein sequence, the C, T, and D indices calculate the 21, 21, and 105 features, respectively. Thus, in this study, a feature vector with the dimension of 147 is obtained, which represents the given protein sequence. The obtained feature vector is normalized in the range [0–1] and obtained the final QLC:


(6) \begin{equation*} QL{C}_j=\frac{x_k-\overline{x}}{std(x)} \end{equation*}

where ${x}_k$ signifies the physiochemical features value of $k- th$ amino acid residue, $\overline{x}$ represents the mean value, $std(x)$ signifies the standard deviation from the mean of 20 amino acids and $QL{C}_j$ represents the obtained QLC vector.

### Sequence encoding using ESM

Recently, inspired by the great success of transformer-based language models in natural language processing, researchers have successfully adapted them to protein sequences [49–53]. By training these language models on enormous data of protein sequences, they aim to further improve the representations of proteins. These protein language models (pLMs) have the ability to learn the contextualized representations (embeddings) within protein sequences that hold significant promise for various challenges related to understanding protein functions. pLMs can efficiently capture the informative details considering both the amino acids (local information) and how they interact within the whole sequence (global information).

**Figure 1:**
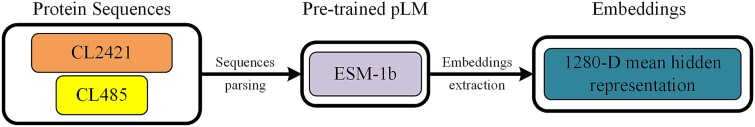
Protein sequence encoding using the protein language model ESM-1b.

Evolutionary scale modeling (ESM) [54, 55] is a powerful pLM based on the architecture of the BERT transformer [56]. The ESM model is trained for a wide and comprehensive understanding of proteins. Numerous researchers have effectively used the ESM model as a feature encoding scheme in their protein-related tasks [33, 57, 58]. Different variants of the ESM model are available based on factors including datasets and number of parameters. In this work, we used an ESM model called esm1b_t33_650M_UR50S (referred as ESM-1b) which is trained on the UniRef50 [59] and has ~650 million learnable parameters. First, we created a FASTA file for each dataset. Each FASTA file was then provided to the ESM-1b to learn and extract the embeddings from each protein sequence. The embeddings of the last hidden layer of the ESM-1b are extracted in this study which has the size of $1280\times L$for each protein sequence, where $L$ is the protein sequence length. We then employed the global average pooling to generate the final $1\times 1280$ dimensional of feature vector representing each protein sequence. Figure 1 illustrates a schematic architecture of the whole process.

### Learning weights for integrating multi-view features

It is commonly believed that the extracted individual features might potentially hold distinct yet complementary information for predicting clathrin proteins. One of the most essential steps in developing a computational model is how to efficiently combine the individual extracted features. The most straightforward method researchers use is the simple serial and direct integration of these individual features to construct a feature set containing multi-information (i.e. ESM+PSSM-CLBP+QLC+RECM-CLBP, where the sign ‘+’ means the serial integration) [60, 61]. However, serial integration ignores the relative importance of the extracted features, which might make the prediction performance of the fused feature set rather inferior to the individual feature set performance. Elucidating the relative importance of the extracted features can significantly improve the predictive accuracy of the clathrin proteins. Therefore, in this study, we adopted the DE algorithm [62, 63] for feature integration due to its high robustness and easy-to-implement nature [64]. Besides, it proved to have a positive impact on a wide range of applications such as signal processing [65], sensor network localization [65], and bioinformatics [66]. The DE algorithm assigns the optimal weights to individual extracted features through a weighted integration strategy. The details to learn the optimum weights of the extracted features are as follows:

Let $f\left(\boldsymbol{w}\right)$ be the objective optimization problem, which is to find the maximum MCC of the *k*-fold cross-validation (CV) test on the CL2421. $w$, which is denoted as $\boldsymbol{w}={\left({w}_1,{w}_2,{w}_3,{w}_4\right)}^T$, is one candidate solution, where each element of $\boldsymbol{w}$ represents the weight assigned to each vector i.e. ${w}_1$ for ESM, ${w}_2$ for PSSM-CLBP, ${w}_3$ for QLC, and ${w}_3$ for RECM-CLBP. All weights of $\boldsymbol{w}$ are bounded within the range of $\left[-2,2\right]$ i.e. $\boldsymbol{w}={\left({w}_1,{w}_2,{w}_3,{w}_4\right)}^T\in{\left[-2,2\right]}^4$. Then, each step can be described as:

#### Initialization

Randomly generate the initial population ${P}^g=\{{\boldsymbol{w}}_1^g,{\boldsymbol{w}}_2^g,{\boldsymbol{w}}_3^g,...,{\boldsymbol{w}}_N^g\}$, where ${\boldsymbol{w}}_j^g={({w}_{j,1}^g,{w}_{j,2}^g,{w}_{j,3}^g,{w}_{j,4}^g)}^T$ is the $j- th$ solution of the $g- th$ generation population and $N$ is the size of the population. Set the parameters of population size ($N$), maximum generations (${G}_{\text{max}}$), crossover rate ($CR$), and scaling factor ($F$) to 100, 50, 0.5, and 0.5, respectively.

#### Mutation

The step of the Mutation is introduced to achieve variation in the search space. Within the population ${P}^g$, for each candidate solution ${\boldsymbol{w}}_j^g$, generate a mutant vector ${\boldsymbol{w}}_j^{g+1}$ using the following equation 6:


(7) \begin{equation*} {\text{v}}_j^{g+1}={\boldsymbol{w}}_{r_1}^g+F\cdotp \left({\boldsymbol{w}}_{r_2}^g-{\boldsymbol{w}}_{r_3}^g\right) \end{equation*}

where ${\boldsymbol{w}}_{r_1}^g$, ${\boldsymbol{w}}_{r_2}^g$ and ${\boldsymbol{w}}_{r_3}^g$ denote three different candidate solutions randomly selected in subset ${P}^g-\{{\boldsymbol{w}}_j^g\}$.

#### Crossover

In order to prevent premature convergence, the DE algorithm introduces the step of crossover to enhance the diversity in the subsequent generation ${P}^{g+1}$. By combining elements from ${\boldsymbol{w}}_j^g$ in ${P}^g$ and ${\boldsymbol{v}}_j^{g+1}$, generate a trial vector ${\boldsymbol{u}}_j^{g+1}={({u}_{j,1}^{g+1},{u}_{j,2}^{g+1},{u}_{j,3}^{g+1},{u}_{j,4}^{g+1})}^T$ as follows:


(8) \begin{equation*} {\boldsymbol{u}}_{j,k}^{g+1}=\left\{\begin{array}{@{}l}{\boldsymbol{v}}_{j,k}^{g+1},\kern1.5em if\ {{rand}}\left(0,1\right)< CR\ or\ k={k}_{rand}\\{}{\boldsymbol{w}}_{j,k}^g,\kern1.5em otherwise\end{array}\right. \end{equation*}

where ${k}_{rand}$ is the randomly selected index of $\left\{1,2,3,4\right\}$ i.e. ${k}_{rand}\in \left\{1,2,3,4\right\}$.

#### Selection

Based on the fitness function $f\left(\cdot \right)$, compare the $f({\boldsymbol{u}}_j^{g+1})$ of the trail vector ${\boldsymbol{u}}_{j,k}^{g+1}$ with the $f({\boldsymbol{w}}_j^g)$of the target solution ${\boldsymbol{w}}_j^g$. If ${\text{u}}_j^{g+1}$ has better fitness value than ${\boldsymbol{w}}_j^g$, ${\boldsymbol{u}}_j^{g+1}$ is set to be ${\boldsymbol{w}}_j^{g+1}$ in the next generation ${P}^{g+1}$, otherwise, ${\boldsymbol{w}}_j^g$ is set to ${\boldsymbol{w}}_j^{g+1}$:


(9) \begin{equation*} {\boldsymbol{w}}_j^{g+1}=\left\{\begin{array}{@{}l}{\boldsymbol{u}}_j^{g+1},\kern0.75em if\ f\left({\boldsymbol{u}}_j^{g+1}\right)>f\left({\boldsymbol{w}}_j^g\right)\\{}{\boldsymbol{w}}_j^g,\kern1.25em otherwise\end{array}\right. \end{equation*}

#### Termination

Terminate the algorithm if $g>{G}_{\text{max}}$and output the best solution ${\boldsymbol{w}}_{best}$ in the population ${P}^g$, otherwise, repeat steps 2 to 4.

In this study, we obtained the best weights as ${\boldsymbol{w}}_{best}=\left(-1.1776,1,1.1734,0.1735\right)$, where the weights of ESM, PSSM-CLBP, QLC, and RECM-CLBP are −1.1776, 1, 1.1734, 0.1735, respectively. Finally, we can generate the integrated feature set (*w*ESM+*w*PSSM-CLBP+*w*QLC+*w*RECM-CLBP) by weightedly integrating ESM, PSSM-CLBP, QLC, and RECM-CLBP.

### Feature selection using BTG algorithm

Feature selection (FS) is one of the most essential techniques in pattern recognition and machine learning tasks. It demonstrably enhances the predictive performance of the prediction models by eliminating redundant, noisy, and irrelevant features from the original features. To further optimize the prediction performance of the clathrin proteins, we leverage a powerful FS algorithm called the binary tree growth (BTG) algorithm [67]. BTG selects the optimal feature subset from the obtained weighted feature set i.e. *w*ESM+*w*PSSM-CLBP+*w*QLC+*w*RECM-CLBP. It is a binary variant of the tree growth algorithm [68]. Complete details of the BTG and FS process, used in this study, can be found in SI Text S5.

### Implementation of SnBiLSTM

In this work, we designed and implemented an SnBiLSTM network as a classification algorithm. The complete details and optimal configuration of the hyperparameters are provided in supplementary Text S6 and Table S6 in SI.

### Architecture of TargetCLP

**Figure 2:**
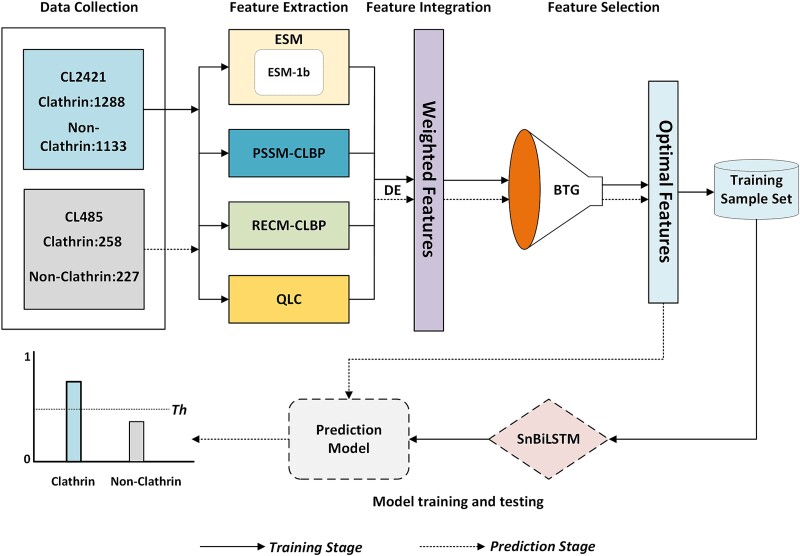
The architecture of the proposed TargetCLP.

Figure 2 demonstrates a diagrammatic overview of the proposed TargetCLP. For a given input protein sequence, by calling the corresponding feature extraction algorithms, TargetCLP extracted ESM, PSSM-CLBP, QLC, and RECM-CLBP features (Feature Extraction). Next, the weights for extracted features are learned using the DE algorithm on the CL2421, and based on the best output weight ${\boldsymbol{w}}_{best}$ the weighted feature integration process is performed to generate a more powerful and discriminative feature vector (Feature Integration). Then, the BTG algorithm is employed to select an optimal subset from the obtained weighted feature set that represents the final feature vector for each protein (Feature Selection). In the training stage, after extracting the final features for all the protein sequences in the benchmark training dataset CL2421, the complete training set is obtained. Finally, the designed snBiLSTM classification algorithm is employed to train the prediction model for targeting clathrin proteins. In the prediction stage, for each given input protein sequence to be predicted, after using the same feature extraction, feature integration, and feature selection steps to generate the final feature vector, the trained prediction model can be used to predict the probability of classifying it to be a clathrin protein. The final prediction is performed based on the predicted probability and predefined threshold $Th$: a protein with a probability larger than the $Th$ is marked as a clathrin protein. The value of the $Th$ is set to 0.5 in this study.

### Performance evaluation metrics

To assess the performance of TargetCLP, in this study, we used the following four classical evaluation metrics of binary classification i.e. Accuracy($Acc$), Sensitivity ($Sen$), Specificity ($Spe$), and Mathew’s Correlation Coefficient ($MCC$). Supplementary Text S7 in SI details the mathematical representation for all the above metrics. We also evaluated the performance by using two essential metrics known as AUPR (Area Under the Precision-Recall Curve) and the AUC (Area Under the Receiver Operating Characteristic [ROC] Curve) which can further assess how well the proposed model distinguishes between the negative and positive samples on a wide level.

Next, to further assess how well the proposed model performs on unseen data, various strategies are used. Among them, the stratified *k*-fold CV and the independent testing are widely used techniques. Therefore, in this study, we also employed stratified *k*-fold CV to prevent overfitting and increase the model’s ability towards unseen data, and the independent dataset, which the model has never encountered in the training stage, is used to judge the model generalization ability.

## Results and discussion

### Performance comparison of individual single-view features

In this subsection, we will evaluate the performance of the individual single-view features, including PSSM-CLBP, RECM-CLBP, QLC, and ESM using various predictive classifiers i.e. CNN, gated recurrent unit (GRU), LSTM, BiLSTM, and SnBiLSTM. Each feature set is evaluated using stratified 5-fold CV on the training dataset CL2421 with five performance indexes of binary classification, including *Acc*, *Sen*, *Spe*, *MCC,* and AUC. Table 1 summarizes the predictive performance comparison of individual single-view features.

**Table 1:** Prediction analysis of individual single-view features using training samples.

Feature	Classifier	*Acc* (%)	*Sen* (%)	*Spe* (%)	*MCC*	AUC
PSSM-CLBP	SnBiLSTM	90.35	92.91	87.98	0.80	0.94
	BiLSTM	89.78	93.74	85.83	0.79	0.92
	LSTM	87.72	89.01	86.57	0.76	0.90
	GRU	85.97	87.01	85.15	0.73	0.89
	CNN	86.02	84.11	88.04	0.72	0.90
	SnBiLSTM	92.57	93.38	91.86	0.85	0.95
	BiLSTM	89.58	91.47	87.80	0.79	0.92
RECM-CLBP	LSTM	86.59	80.64	92.12	0.74	0.89
	GRU	84.11	83.10	84.85	0.68	0.88
	CNN	84.63	86.27	83.09	0.69	0.88
QLC	SnBiLSTM	91.69	93.50	89.95	0.83	0.94
	BiLSTM	89.32	88.91	89.99	0.78	0.92
	LSTM	86.69	86.33	87.21	0.73	0.90
	GRU	82.21	74.23	90.45	0.66	0.89
	CNN	86.12	86.83	85.59	0.72	0.90
ESM	SnBiLSTM	91.80	94.22	89.46	0.83	0.94
	BiLSTM	89.37	89.75	89.22	0.79	0.92
	LSTM	89.07	89.01	89.28	0.78	0.92
	GRU	88.25	89.69	87.07	0.77	0.91
	CNN	87.16	88.65	85.81	0.74	0.90

Several observations can be concluded from Table 1. First, comparing the individual features, the proposed RECM-CLBP has an outstanding prediction performance on the SnBiLSTM with an *Acc* = 92.57%*, MCC* = 0.85, and AUC = 0.95. ESM embeddings achieved the second-best predictive performance in terms of *Acc* = 91.80%, however, in terms of *MCC* the QLC and ESM achieved similar performances of 0.83 and 0.83, respectively, on the snBiLSTM. Similarly, in terms of AUC, QLC, ESM, and the proposed PSSM-CLBP achieved a similar performance of 0.94 on the SnBiLSTM. From this observation, we can conclude that all four individual single-view features have better predictive performance on the training dataset; Second, comparing the performance of CNN, GRU, LSTM, BiLSTM, and SnBiLSTM using the four individual single-view features, the SnBiLSTM achieved the best performance on all the features. The BiSLTM achieved the second-best performance with *Acc* of 89.78%, 89.58%, 89.32%, and 89.37% and *MCC* of 0.79, 0.79, 0.78, 0.79 on the PSSM-CLBP, RECM-CLBP, QLC, and ESM, respectively. Similarly, the LSTM, GRU, and CNN also achieved favorable performance on all four features. This performance compression suggests that the SnBiLSTM is more powerful than the remaining well-known classifier algorithms. Third, altogether, we can find that both the proposed PSSM-CLBP and RECM-CLBP achieved high performance. This is because the CLBP captures more intrinsic information in the PSSM and RECM matrices. Additionally, Table 1 also suggests that the ESM embedding is important for predicting clathrin proteins.

**Figure 3:**
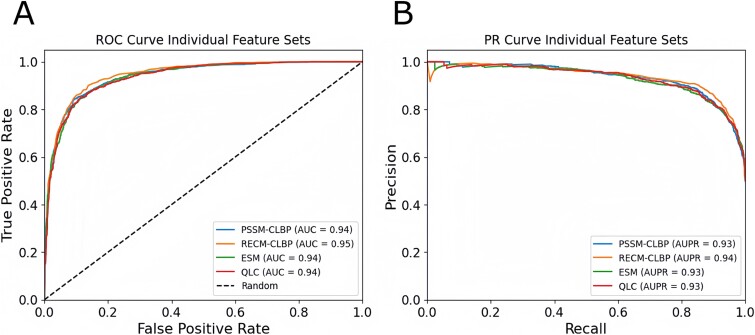
ROC and PR curves of individual features using SnBiLSTM classifier: Panels (A) and (B) show the ROC and PR curves, respectively.

To further show the effect of each feature on how well the individual features distinguish positive and negative samples, we calculated the ROC and PR curves for each feature set using the SnBiLSTM, and the outcome is provided in Fig. 3. Figure 3A and B show the ROC and PR curves, respectively. We can see that all four individual features achieve consistent performance, once again, validating that these features are important for distinguishing clathrin proteins from non-clathrin proteins.

### Weighted feature integration can enhance the model performance

In order to effectively verify the predictive ability of the weighted feature integration, in this subsection, we compare the prediction performance of ESM+PSSM-CLBP+QLC+RECM-CLBP and *w*ESM+*w*PSSM-CLBP+*w*QLC+*w*RECM-CLBP using stratified 5-fold CV on the training dataset CL2421 with five different classifiers. The performance outcome is summarized in Table 2.

**Table 2:** Performance comparison of serially and weightedly integrated features on the training dataset.

Dataset	Method	Classifier	*Acc* (%)	*Sen* (%)	*Spe* (%)	*MCC*	AUC
Training Dataset	S-Features^a^	SnBiLSTM	92.52	92.23	92.78	0.85	0.95
		BiLSTM	90.30	90.67	89.89	0.80	0.92
		LSTM	90.14	90.07	90.30	0.80	0.92
		GRU	88.19	89.04	87.21	0.76	0.90
		CNN	89.06	88.53	89.44	0.78	0.92
	W-Features^b^	SnBiLSTM	93.65	93.14	95.42	0.89	0.96
		BiLSTM	91.49	90.50	92.48	0.83	0.94
		LSTM	91.12	90.38	91.83	0.82	0.93
		GRU	89.58	88.05	91.15	0.79	0.91
		CNN	90.02	89.78	92.26	0.82	0.92

^a^S-Features mean the ESM+PSSM-CLBP+QLC+RECM-CLBP feature set.

^b^W-Features mean the *w*ESM+*w*PSSM-CLBP+*w*QLC+*w*RECM-CLBP feature set.

From the performance results in Table 2, we can clearly observe that the feature set using weighted-based feature integration, i.e. *w*ESM+*w*PSSM-CLBP+*w*QLC+*w*RECM-CLBP consistently outperforms the feature set using simple serial integration strategy, i.e. *-*ESM+PSSM-CLBP+QLC+RECM-CLBP on all the five classification algorithms, concerning all the five performance measure indexes. Particularly, on the best prediction algorithm SnBiLSTM, the *Acc, MCC,* and AUC of *w*ESM+*w*PSSM-CLBP+*w*QLC+*w*RECM-CLBP are 93.65%, 0.89, 0.96, which are 1.21%, 4.59% and 1.04% higher than ESM+PSSM-CLBP+QLC+RECM-CLBP, respectively. Similarly, the *w*ESM+*w*PSSM-CLBP+*w*QLC+*w*RECM-CLBP also achieved superior performance on the second-best classifier BiLSTM in comparison to the ESM+PSSM-CLBP+QLC+RECM-CLBP, concerning all the performance measure indexes.

By revising and comparing Table 1 and Table 2, we can reveal a noteworthy observation that is the *Acc* of RECM-PSSM is slightly higher than the features integrated in serial integration strategy, i.e. ESM+PSSM-CLBP+QLC+RECM-CLBP, on the best classifier SnBiLSTM. This phenomenon concludes that the serial integration of these individual single-view features, i.e. PSSM-CLBP, RECM-CLBP, QLC, and ESM cannot improve the prediction performance of the clathrin proteins. While comparing to RECM-PSSM, the *w*ESM+*w*PSSM-CLBP+*w*QLC+*w*RECM-CLBP can achieve higher predictive performance on the best-performing classifier SnBiLSTM. Together, the findings from the above comparison suggest potential benefits to using weighted-based feature integration.

### Improving prediction performance by FS

Our findings, presented in the previous sections, clearly revealed that weighted feature integration of the individual single-view features i.e. PSSM-CLBP, RECM-CLBP, QLC, and ESM, improves the performance of clathrin proteins prediction. The total feature dimension of these weighted-based integration features is 1899, which includes 1280, 236, 147, and 236 features of ESM, PSSM-CLBP, QLC, and RECM-CLBP, respectively. To mitigate the overfitting risk, improve the prediction performance, and further enhance the proposed method’s ability towards the unseen clathrin data, an effective feature selection algorithm can play a significant role. Therefore, we applied the BTG algorithm on the *w*ESM+*w*PSSM-CLBP+*w*QLC+*w*RECM-CLBP feature set and obtained a subset with 969-dimension of optimal features and the performance was evaluated on different classifiers using stratified 5-fold CV on the training dataset. Table 3 summarizes the predictive performance after employing BTG on the *w*ESM+*w*PSSM-CLBP+*w*QLC+*w*RECM-CLBP feature set.

**Table 3:** Prediction outcomes with BTG feature selection algorithm using the training dataset.

Dataset	Method	Classifier	*Acc* (%)	*Sen* (%)	*Spe* (%)	*MCC*	AUC
Training Dataset (CL2421)	W-Features +BTG^a^	SnBiLSTM	95.37	94.70	95.96	0.90	0.98
		BiLSTM	93.39	93.97	92.83	0.86	0.96
		LSTM	92.41	92.73	92.10	0.84	0.95
		GRU	91.23	90.41	92.09	0.82	0.94
		CNN	91.12	90.07	92.19	0.82	0.94

^a^W-Features+BTG means feature set obtained after BTG.

From the given results in Table 3, we can see that after applying BTG, the SnBiLSTM classification algorithm achieved the *Acc*, *MCC,* and AUC of 95.37%, 0.90, and 0.98, respectively, followed by BiLSTM with *Acc* = 93.97, *MCC* = 0.86 and AUC = 0.96. By revisiting Table 2 and comparing the results to *w*ESM+*w*PSSM-CLBP+*w*QLC+*w*RECM-CLBP, we can observe that the prediction performance after employing BTG on *w*ESM+*w*PSSM-CLBP+*w*QLC+*w*RECM-CLBP consistently improved on all classifiers concerning five evaluation indexes. Concretely, after employing the BTG algorithm, the *Acc*, *MCC,* and AUC are increased by 1.82%, 1.11%, and 2.06%, respectively, over *w*ESM+*w*PSSM-CLBP+*w*QLC+*w*RECM-CLBP on the SnBiLSTM which shows the effectiveness of using BTG FS algorithm. Similarly, in terms of *Sen* and *Spe*, the SnBiLSTM also showed improvements. Comparing the remaining four classifiers in Table 2 and Table 3, i.e. BiLSTM, LSTM, GRU, and CNN, the reduced features, employing BTG, showed improvements in terms of all five evaluation indexes over the high dimension *w*ESM+*w*PSSM-CLBP+*w*QLC+*w*RECM-CLBP which again proves the significant importance of using effective feature selection algorithm.

**Figure 4:**
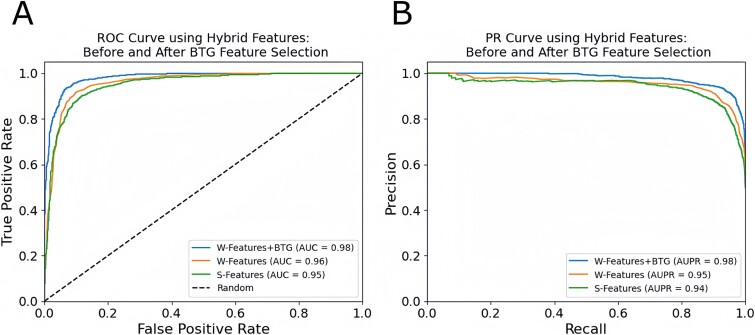
ROC and PR curves of integrated features using SnBiLSTM classifier: Panels (A) and (B) show the ROC and PR curves, respectively, for S-Features, W-Features, and W-Features+BTG.

To further show the performance in terms of ROC and PR curves, we summarized the results in Fig. 4, where Fig. 4A and B show the ROC and PR curves of S-Features, W-Features, and W-features+BTG, respectively, using the SnBiLSTM classifier. When comparing, the W-Features, which is the weighted-based integration, outperformed the S-Features, which is the serial integration of four single-view features, in terms of both ROC and PR curves. Similarly, the W-Features+BTG achieved the best performance than the S-Features and W-features. Regarding AUC, as we discussed earlier, however, looking at the AUPR values in Fig. 4B, which are 0.98, 0.95, and 0.94 for W-Features+BTG, W-Features, and S-Features, respectively, we can observe that both the W-Features and W-Features+BTG achieved better performance than the S-Features while the W-Features+BTG is the best performer.

In addition, to evaluate the model prediction performance, the Unified Manifold Approximation and Projection [69] and the SHaply Additive exPlanation (SHAP) [70] analyses were performed to map the extracted features for the targeted classes and understand how the individual features in the selected optimal vectors contribute to the prediction performance of the model. The details and discussion are provided in the SI Text S8 and Text S9.

### Comparison of various classifiers using CL485 independent dataset

In this subsection, we will show the generalization ability using various classification algorithms. Therefore, we trained each of the classification algorithms, i.e. SnBiLSTM, BiLSTM, LSTM, GRU, CNN, on the whole training dataset CL2421 using the obtained reduced optimal features after employing the BTG algorithm and tested the performance on the unseen independent dataset CL485. The results outcomes achieved on all the classifiers are summarized in Table 4.

**Table 4:** Prediction outcomes of various classifiers using independent dataset.

Dataset	Method	Classifier	Acc (%)	Sen (%)	Spe (%)	MCC	AUC
Independent Dataset (CL485)	W-Features +BTG	SnBiLSTM	92.78	89.42	95.72	0.85	0.96
		BiLSTM	91.75	87.69	95.33	0.83	0.94
		LSTM	90.72	85.50	95.33	0.81	0.92
		GRU	90.51	85.48	94.96	0.81	0.92
		CNN	89.88	86.20	92.37	0.78	0.91

**Figure 5:**
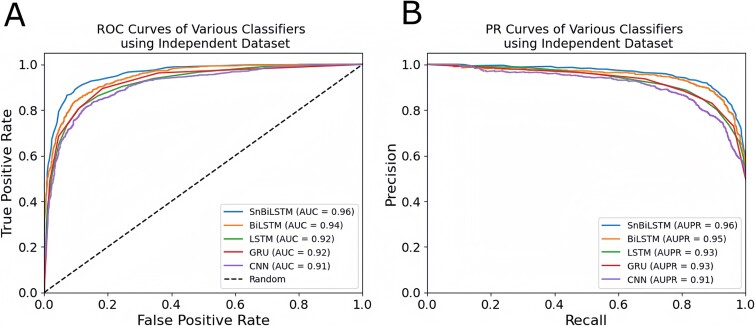
ROC and PR curves of various classifiers using independent dataset: Panels (A) and (B) show the ROC and PR curves, respectively.

We can observe in Table 4 that all the classifiers have favorable and competitive performance in terms of all evaluation indexes. This significant performance shows better generalization capability over all the trained classifiers. However, when comparing the classifiers, SnBiSLTM once again showed tremendous predictive performance in terms of generalization ability over all the evaluation metrics. Specifically, the *Acc* = 92.78, *MCC* = 0.85, and AUC = 0.96 of snBiLSTM are improved by 3.17%, 8.58% and 5.34 on the CNN; 2.47%, 4.81 and 4.25 on the GRU; 2.24%, 4.81 and 4.25 on the LSTM; 1.11%, 2.38 and 2.10 on the BiLSTM, respectively. Similarly, in terms of *Sen* and *Spe*, the SnBiLSTM has better performance than the other classifiers. Again, we show the effect in terms of ROC and PR curves in Fig. 5, which shows more consistent curves and values for both ROC in Fig. 5A and PR in Fig. 5B, using SnBiLSTM. This further shows that the SnBiLSTM is the best prediction classifier for predicting clathrin proteins.

As SnBiLSTM consistently achieved the best performance on the benchmark training dataset using stratified 5-fold CV as well as better generalization ability on the independent dataset, we will train our final TargetCLP predictor using the SnBiLSTM classification algorithm.

### Comparison of TargetCLP with existing methods on the CL2421 training dataset

This section is devoted to comparing the performance of the TargetCLP with existing work. According to the existing literature, only one computational-based predictor, i.e. Le et al. predictor [4], has been developed to date. We performed experiments on the training dataset CL2421 and the results outcomes were summarized in Table 5.

**Table 5:** Performance comparison of TargetCLP with the past work on the benchmark training dataset CL2421.

Predictor	*Acc* (%)	*Sen* (%)	*Spe* (%)	*MCC*	AUC
Le et al. predictor	91.70	91.70	91.60	0.83	–
TargetCLP	95.37	94.70	95.96	0.90	0.98

As shown in Table 5, the predictor proposed by Le et al. produced *Acc* and *MCC* of 91.70% and 0.83 points, however, our method produced *Acc* and *MCC* of 95.37% and 0.90 points, respectively, which demonstrates the effectiveness of our TargetCLP method for clathrin protein prediction. More concretely, our proposed TargetCLP showed a 3.92% improvement using *Acc* and an 8.09% improvement using *MCC* in comparison to the predictive *Acc* and *MCC* of Le et al. predictor, which proves the remarkable performance of TargetCLP. Our proposed predictor also performed better in terms of other evaluation indexes. Part of the results in Table 5 is excerpted from Le et al.’s work [4], where they did not calculate the performance of the model in terms of AUC. However, we calculated the AUC of our proposed method to show the strength of the model as well as help the researchers easily compare their works in the future. Together all the results in Table 5 show that the TargetCLP achieved remarkable performance over the existing state-of-the-art predictor on the training dataset.

### Comparison of TargetCLP with existing methods on the CL485 independent dataset

**Figure 6:**
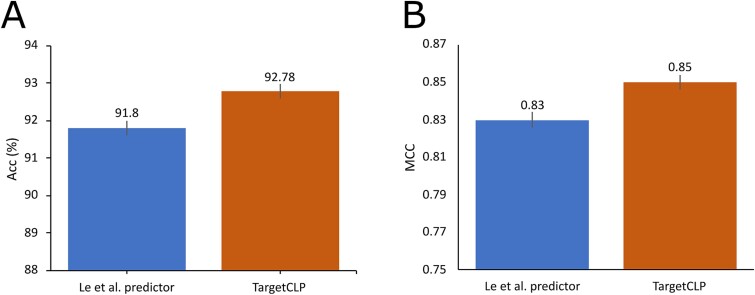
Performance comparison of TargetCLP and Le et al. predictor: Panel (A) shows the performance in term of Acc while panel (B) shows the performance in term of MCC.

To further validate the generalization ability of the TargetCLP, we trained TargetCLP on the training dataset CL2481 and tested it on the independent dataset CL485. We calculated the performance in terms of all five evaluation indexes. The performance results of the Le et al. predictor, which are excerpted from their work, and our TargetCLP are recorded in Fig. 6A and B and Table S7 under Text S10 in SI, which clearly outperformed the Le et al. method in terms of generalization power. Here, Fig. 6A and B is provided to facilitate an easy understanding of the effectiveness and generalization ability of the proposed TargetCLP against the existing state-of-the-art predictor on the independent dataset.

While the key goal of the proposed study was to boost the predictive performance and interpretability, we acknowledge that computational efficacy and scalability are critical using TargetCLP for larger datasets. we have included a brief discussion in the SI Text S11, highlighting this issue.

## Conclusion

Accurate identification of clathrin proteins is crucial for understating their functional roles and regulatory mechanisms. To improve the performance of clathrin protein prediction, we proposed a novel and efficient clathrin protein predictor, named TargetCLP. Concretely, in the first step, TargetCLP extracted single-view ESM, PSSM-CLBP, RECM-CLBP, and QLC feature vectors for each sequence. All four single-view feature vectors were weightedly integrated using DE and the BTG feature selection algorithm was employed to obtain a multiview optimal subset for each protein sequence. The obtained optimal set was fed into SnBiLSTM to train the TargetCLP. Benchmark experiments using CL2421 and CL485 showed that the TargetCLP achieved remarkable performance than existing state-of-the-art work in the literature. The high performance and generalization ability of the TargetCLP lies in the (i) vigilant numerical representation of the protein sequence with ESM, PSSM-CLBP, and RECM-CLBP; (ii) integrating single-view numerical representations with DE to obtain improved and optimized multi-view feature set, and (iii) the careful training and optimization process of the self-normalized based BiLSTM i.e. SnBiLSTM.

Although TargetCLP achieved the best predictive performance by incorporating advanced feature engineering and pLM models, there is still room for further advancement in the model. The ESM embeddings, which effectively capture residue-level features, do not consider the entire protein with a size larger than its limit. Moreover, this study does not explicitly consider the computational complexity of the model. The current model only focused on the dataset with limited scope. To further enhance the performance, the following aspects will be considered in the future: (i) Up-to-date and larger datasets will be collected for large-scale prediction; (ii) exploring new techniques for sequence embeddings; and (iii) explicitly considering the computational complexity of the model. In addition, we haven’t found a user-friendly web server in the literature; therefore, there is a need for a user-friendly web server as well. While TargetCLP has potential for further improvement, it currently stands as a valuable method for identifying clathrin proteins.

**Key Points**

- The study proposed transformed image-based features and integrated them with ESM embeddings for generating enhanced feature vectors.
- The weight for each feature set was learned using a differential evolution algorithm for integrating multi-view feature sets via a weighted feature integration strategy.
- The Binary tree algorithm was implemented to reduce and select the optimal feature set from the obtained weighted feature set.
- Based on the developed pipeline, A novel computational method, TargetCLP, trained on the designed SnBiLSTM network, was implemented. Experimental results on both training and testing datasets verified the remarkable performance of TargetCLP.
- The high performance of the TargetCLP is due to the vigilant numerical representation of the protein sequences, the integration of features with DE, training, and optimization of the SnBiLSTM model.

## Supplementary Material

SI_bbaf026

## Data Availability

All the data and source codes utilized in this study are freely accessible at https://github.com/MateeullahKhan/TargetCLP.
