# Supplementary material for: TargetCLP: clathrin proteins prediction combining transformed and evolutionary scale modeling-based multi-view features via weighted feature integration approach
Source: Brief Bioinform. 2025 Jan 23;26(1):bbaf026. doi: 10.1093/bib/bbaf026 (PMC11753890; doi:10.1093/bib/bbaf026)
Supplement: SI_bbaf026 [file si_bbaf026.docx]

**Supporting Information**

**TargetCLP: clathrin proteins prediction combining transformed and evolutionary scale modeling-based multi-view features via weighted feature integration approach**

Matee Ullah^1^, Shahid Akbar^1,2^, Ali Raza^3^, Kashif Ahmad Khan^2^, Quan Zou^1,4,*^

^1^Institute of Fundamental and Frontier Sciences, University of Electronic Science and Technology of China, Chengdu 610054, Sichuan, China.

^2^Department of Computer Science, Abdul Wali Khan University Mardan, Mardan 23200, Pakistan.

^3^Department of Computer Science, MY University, Islamabad 45750, Pakistan.

^4^Yangtze Delta Region Institute (Quzhou), University of Electronic Science and Technology of China, Quzhou, 324003, Zhejiang, China.

*To whom correspondence should be addressed.

# **Text S1. Statistical Analysis of the Training and Independent Datasets**

We have conducted an independent analysis of the training and testing dataset using the DeepLoc 2.0 to further show the subcellular localization of the Clathrin and non-Clathrin proteins. The subcellular localization of each protein was analyzed to provide context on its potential roles within the cell. This analysis can help refine the understanding of the biological significance in which these proteins function, provide guidance for further experimental studies.

The DeepLoc 2.0 only accommodates proteins with minimum and maximum lengths of 10 and 6000, respectively. We have analyzed that in the training dataset, one Clathrin protein is short with length below 10 and two of the proteins’ length (1 Clathrin and one Non-Clathrin Proteins) are longer than the maximum limit of the DeepLoc 2.0. Therefore, we removed those three proteins from our training data only for analyzing with DeepLoc 2.0. The Statistical analysis for the Clathrin and non-Clathrin proteins in the training dataset are provided in Table S1, while Table S2 shows the statistical analysis for the independent dataset.

**Table S1.** Statistical Summary of the predicted subcellular localization of the training dataset using DeepLoc 2.0

| **Clathrin Proteins (1286)** | | | | **Non-Clathrin Proteins (1132)** | | | |
| --- | --- | --- | --- | --- | --- | --- | --- |
| **Subcellular location** | **Single-Label Localizations** | **Multi-Label Localizations** | | **Subcellular location** | **Single-Label Localizations** | **Multi-Label Localizations** | |
| Cytoplasm | 307 | 358 | | Cytoplasm | 187 | 159 | |
| Nucleus | 37 | 111 | | Nucleus | 47 | 73 | |
| Extracellular | 49 | 7 | | Extracellular | 70 | 3 | |
| Cell membrane | 254 | 163 | | Cell membrane | 110 | 163 | |
| Mitochondrion | 4 | 0 | | Mitochondrion | 11 | 4 | |
| Plastid | 0 | 0 | | Plastid | 1 | 0 | |
| Endoplasmic reticulum | 94 | 39 | | Endoplasmic reticulum | 108 | 106 | |
| Lysosome/ Vacuole | 27 | 207 | | Lysosome/ Vacuole | 60 | 250 | |
| Golgi apparatus | 34 | 146 | | Golgi apparatus | 91 | 195 | |
| Peroxisome | 2 | 1 | | Peroxisome | 1 | 0 | |
| -The total number of Clathrin proteins with Single-Label Localization | | | 808 | -The total number of non-Clathrin proteins with Single-Label Localization are | | | 686 |
| -The total Clathrin proteins with Multi-Label Localization | | | 478 | -The total number of non-Clathrin proteins with Multi-Label Localization | | | 446 |

**Table S2.** Statistical Summary of the predicted subcellular localization of the independent dataset using DeepLoc 2.0

| **Clathrin Proteins (258)** | | | | **Non-Clathrin Proteins (227)** | | | |
| --- | --- | --- | --- | --- | --- | --- | --- |
| **Subcellular location** | **Single-Label Localizations** | **Multi-Label Localizations** | | **Subcellular location** | **Single-Label Localizations** | **Multi-Label Localizations** | |
| Cytoplasm | 71 | 51 | | Cytoplasm | 46 | 27 | |
| Nucleus | 23 | 17 | | Nucleus | 5 | 17 | |
| Extracellular | 18 | 0 | | Extracellular | 10 | 2 | |
| Cell membrane | 31 | 42 | | Cell membrane | 20 | 28 | |
| Mitochondrion | 1 | 1 | | Mitochondrion | 1 | 3 | |
| Plastid | 0 | 0 | | Plastid | 0 | 1 | |
| Endoplasmic reticulum | 12 | 5 | | Endoplasmic reticulum | 36 | 16 | |
| Lysosome/ Vacuole | 4 | 54 | | Lysosome/ Vacuole | 9 | 35 | |
| Golgi apparatus | 4 | 29 | | Golgi apparatus | 28 | 23 | |
| Peroxisome | 0 | 3 | | Peroxisome | 0 | 1 | |
| -The total number of Clathrin proteins with Single-Label Localization | | | 164 | -The total number of non-Clathrin proteins with Single-Label Localization are | | | 155 |
| -The total Clathrin proteins with Multi-Label Localization | | | 94 | -The total number of non-Clathrin proteins with Multi-Label Localization | | | 72 |

It is noteworthy that for the multi-Label Localization, one protein can occur more than one location. For example, a Clathrin protein with protein_ID=P18801 is predicted in both Cytoplasm and Cell membrane. Similarly, taking example from the Table S1, the 358 Proteins that are predicted as Cytoplasm, are also predicted in one or more locations. Figure S1(A-B) and Figure S2 (A-B) provide statistical summary of the predicted localization in graphical view for the two datasets.


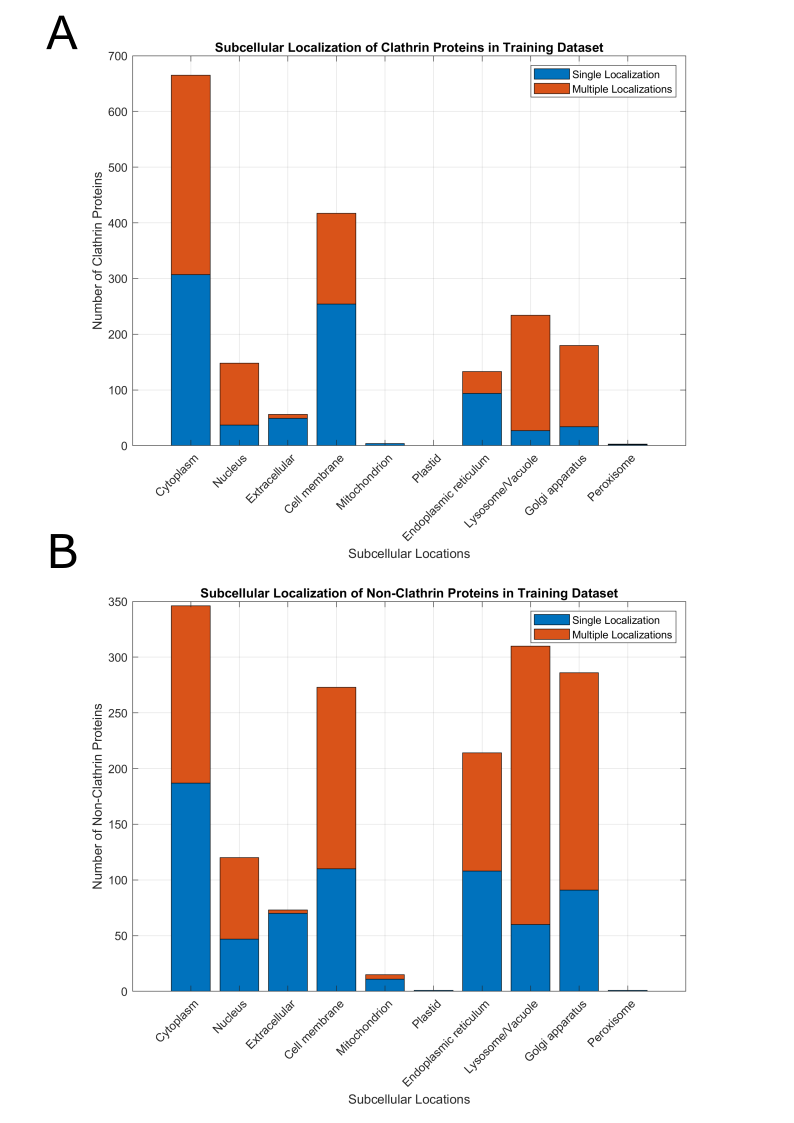


**Figure S1.** The distribution of the clathrin proteins subcellular localization of the training dataset using DeepLoc 2.0. (A) shows the distribution of the clathrin proteins while (B) shows the distribution of the non-clathrin proteins.


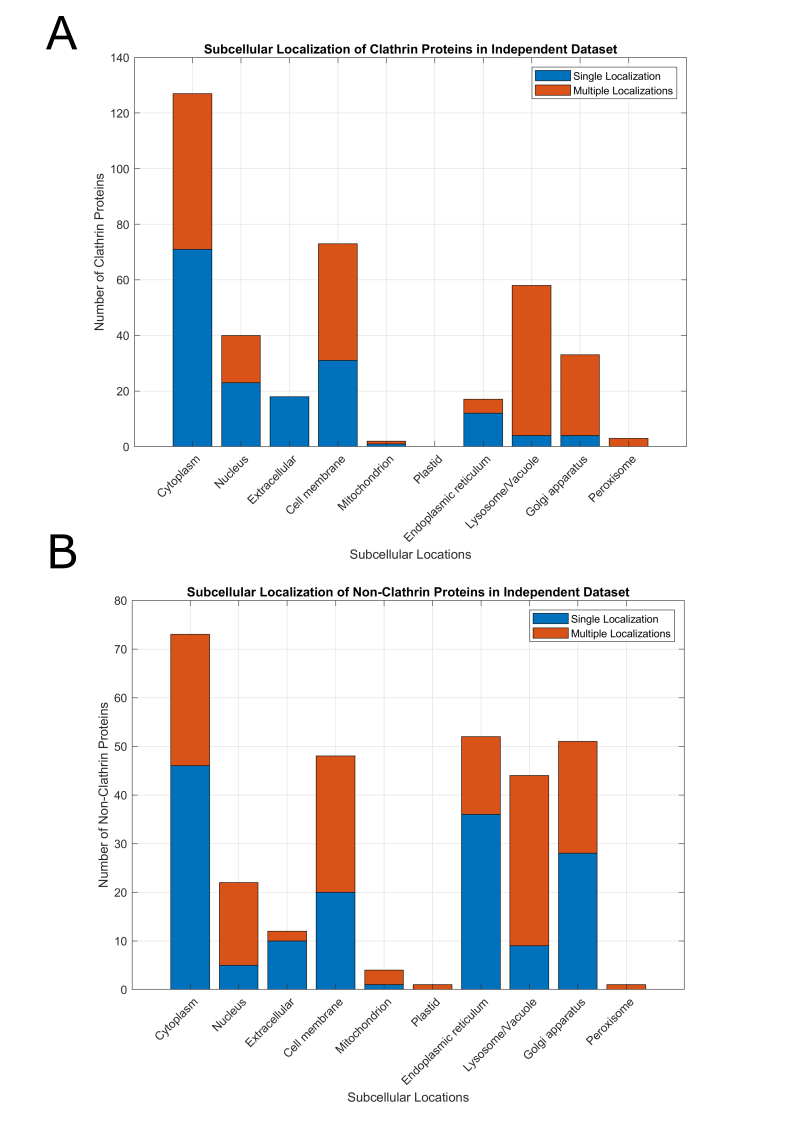


**Figure S2.** The distribtuion of the clathrin proteins subcellular localization of the independent dataset using DeepLoc 2.0. (A) Shows the distribution of the clathrin proteins while (B) shows the distribution of the non-clathrin proteins.

# **Text S2. Residue-wise Energy Contact Matrix**

The following Table S1 is the residue-wise energy contact matrix (RECM) used as an energy contact matrix in this study. The RECM is a $20\times20$ a square matrix whose contact pairwise energies were obtained using the 674 primary protein sequences by least-square fitting with 785 proteins of tertiary structures.

| **Table S3.** The residue-wise energy contact matrix (RECM)used in this study. | -4.62 | -4.46 | 0.9 | 1.29 | -8.8 | -1.9 | -3.2 | -5.26 | -1.19 | -4.9 | -9.73 | 0.93 | -2.09 | 0.01 | 0.36 | -0.82 | -0.37 | -3.59 | -12.39 | -2.68 |
| --- | --- | --- | --- | --- | --- | --- | --- | --- | --- | --- | --- | --- | --- | --- | --- | --- | --- | --- | --- | --- |
|  | 0.32 | 4.26 | -0.71 | -1.07 | -7.09 | 1.69 | -7.58 | -3.78 | 0.02 | -8.31 | -6.88 | -0.74 | -2.06 | -0.76 | -5.89 | -3.03 | -0.65 | -2.13 | -1.73 | -12.39 |
|  | -2.31 | -0.16 | 0.94 | 0.12 | -7.05 | -0.38 | 0.27 | -6.54 | 0.19 | -5.43 | -2.59 | 0.93 | 0.38 | -1.91 | 0.08 | 0.13 | 1.14 | -4.82 | -2.13 | -3.59 |
|  | 0.46 | -1.84 | -0.65 | 1.54 | 0.11 | 0.59 | -0.01 | 0.63 | -1.11 | 0.72 | 0.63 | 0.46 | 1.65 | -0.07 | 0.98 | -0.06 | -0.96 | 1.14 | -0.65 | -0.37 |
|  | -0.08 | -2.33 | 0.91 | 0.81 | -2.22 | 0.71 | 0.82 | -0.15 | 0.19 | -0.41 | 1.39 | 0.29 | 1.12 | 0.85 | 0.95 | -0.48 | -0.06 | 0.13 | -3.03 | -0.82 |
|  | 0.98 | -0.41 | -2.02 | -3.13 | -0.4 | 0.84 | 2.05 | 0.19 | 2.34 | -0.6 | 2.09 | 1.08 | 1.06 | 0.91 | 0.21 | 0.95 | 0.98 | 0.08 | -5.89 | 0.36 |
|  | 1.2 | -2.91 | 2.67 | 0.1 | 0.77 | 1.11 | 2.64 | -0.18 | 0.43 | -0.58 | 1.9 | 1.28 | 2.97 | -1.54 | 0.91 | 0.85 | -0.07 | -1.91 | -0.76 | 0.01 |
|  | 1.54 | -2.13 | 3.31 | 1.44 | 0.32 | 2.25 | 0.35 | 0.12 | 0.51 | 1.81 | 0.75 | 1.15 | -0.42 | 2.97 | 1.06 | 1.12 | 1.65 | 0.38 | -2.06 | -2.09 |
|  | 0.66 | -4.18 | 0.32 | 0.2 | 0.73 | -0.32 | 1.84 | -0.07 | 1.12 | 0.97 | 0.21 | 0.61 | 1.15 | 1.28 | 1.08 | 0.29 | 0.46 | 0.93 | -0.74 | 0.93 |
|  | -2.08 | 1.43 | 0.61 | 2.53 | -5.34 | -0.52 | -0.75 | -3.62 | 1.61 | -2.88 | -6.49 | 0.21 | 0.75 | 1.9 | 2.09 | 1.39 | 0.63 | -2.59 | -6.88 | -9.73 |
|  | -3.01 | -2.15 | 0.23 | 1.14 | -8.59 | -0.55 | -0.86 | -9.01 | 0.49 | -6.37 | -2.88 | 0.97 | 1.81 | -0.58 | -0.6 | -0.41 | 0.72 | -5.43 | -8.31 | -4.9 |
|  | 0.49 | -1.38 | -1.93 | -2.51 | -0.82 | -0.16 | 2.89 | -0.01 | 1.24 | 0.49 | 1.61 | 1.12 | 0.51 | 0.43 | 2.34 | 0.19 | -1.11 | 0.19 | 0.02 | -1.19 |
|  | -3.69 | 0.34 | 0.68 | 1.3 | -5.88 | -0.65 | -0.71 | -6.74 | -0.01 | -9.01 | -3.62 | -0.07 | 0.12 | -0.18 | 0.19 | -0.15 | 0.63 | -6.54 | -3.78 | -5.26 |
|  | 1.9 | -4.98 | -1.07 | 0.61 | -3.57 | 1.09 | 1.97 | -0.71 | 2.89 | -0.86 | -0.75 | 1.84 | 0.35 | 2.64 | 2.05 | 0.82 | -0.01 | 0.27 | -7.58 | -3.2 |
|  | -0.41 | -2.96 | 0.88 | 1.31 | 0.35 | -0.2 | 1.09 | -0.65 | -0.16 | -0.55 | -0.52 | -0.32 | 2.25 | 1.11 | 0.84 | 0.71 | 0.59 | -0.38 | 1.69 | -1.9 |
|  | -3.73 | -3.07 | -0.92 | 0.94 | -11.25 | 0.35 | -3.57 | -5.88 | -0.82 | -8.59 | -5.34 | 0.73 | 0.32 | 0.77 | -0.4 | -2.22 | 0.11 | -7.05 | -7.09 | -8.8 |
|  | 1.8 | -0.53 | 1.97 | 1.45 | 0.94 | 1.31 | 0.61 | 1.3 | -2.51 | 1.14 | 2.53 | 0.2 | 1.44 | 0.1 | -3.13 | 0.81 | 1.54 | 0.12 | -1.07 | 1.29 |
|  | 1.16 | -0.82 | 0.84 | 1.97 | -0.92 | 0.88 | -1.07 | 0.68 | -1.93 | 0.23 | 0.61 | 0.32 | 3.31 | 2.67 | -2.02 | 0.91 | -0.65 | 0.94 | -0.71 | 0.9 |
|  | -2.83 | -39.58 | -0.82 | -0.53 | -3.07 | -2.96 | -4.98 | 0.34 | -1.38 | -2.15 | 1.43 | -4.18 | -2.13 | -2.91 | -0.41 | -2.33 | -1.84 | -0.16 | 4.26 | -4.46 |
|  | -1.65 | -2.83 | 1.16 | 1.8 | -3.73 | -0.41 | 1.9 | -3.69 | 0.49 | -3.01 | -2.08 | 0.66 | 1.54 | 1.2 | 0.98 | -0.08 | 0.46 | -2.31 | 0.32 | -4.62 |

# **Text S3. Working Mechanism of PSSM and RECM into CLBP features**

To understand the basic concept of how we transformed the PSSM and RECM into feature vector using CLBP descriptor, below we provide two examples with 3 x 3 window:

PSSM Matrix Transformation:

Given the first example

| *P_1_* = | 0 | 3 | 0 |
| --- | --- | --- | --- |
|  | 0 | -1 | -2 |
|  | -2 | -1 | -2 |

As noted in the manuscript, the PSSM (the given window here) is first mapped into 0 to 255 range, which is represented as a gray scale image, using min-max normalization in the range of [0-255]. By doing so, we get the following *I(P_1_)* gray scale image:

| *I(P_1_)* = | 102 | 255 | 102 |
| --- | --- | --- | --- |
|  | 102 | 51 | 0 |
|  | 0 | 51 | 0 |

After the matrix is mapped into gray scale image, we then calculate the CLBP_C, CLBP_S and CLBP_M as:

By calculating the average threshold from the above gray scale image, we get 73.67 as the threshold. Since the center pixel is smaller than the average threshold, the CLBP_C will be:

CLBP_C = 0

Similarly, when calculating CLBP_S, the neighbor pixels are compared with the center pixel.

| CLBP_S = | 1 | 1 | 1 |
| --- | --- | --- | --- |
|  | 1 |  | -1 |
|  | -1 | 1 | -1 |

Finally, reading the above matrix in clockwise direction, with negative values replaced by 0, we can get:

CLBP_S = 11001011

For the CLBP_M, we calculate the magnitude difference between the 8 neighbors and center pixel. By calculating we get the magnitude difference as:

| CLBP_M*_difference_* = | 51 | 204 | 51 |
| --- | --- | --- | --- |
|  | 51 |  | 51 |
|  | 0 | 0 | 51 |

Now by calculating either local or global average threshold, we can convert the above magnitude difference matrix into binary form. Assuming the average threshold of the magnitude difference matrix, we can get 56.67. If the value in the magnitude difference is greater than or equal to 56.67 assign 1, otherwise, assign 0. Using this formula, we can convert the magnitude values into binary values as:

| CLBP_M = | 0 | 1 | 0 |
| --- | --- | --- | --- |
|  | 0 |  | 0 |
|  | 0 | 0 | 0 |

Reading clockwise, we get

CLBP_M = 10000000

All the binary values are then converted into decimal equivalent and histogram is calculated to further generate the final feature vector.

Example 2:

By following the same procedure discussed above, we can calculate the CLBP_C, CLBP_S and CLBP_M as:

| *P_2_* = | 8 | 0 | -6 |
| --- | --- | --- | --- |
|  | -2 | 2 | -3 |
|  | 2 | 6 | 5 |

The gray scale transformed image *I(P_2_)* is represented as:

| *I(P_2_)* = | 255 | 109 | 0 |
| --- | --- | --- | --- |
|  | 73 | 146 | 55 |
|  | 146 | 219 | 200 |

For CLBP_C, as the average threshold is 133.67 which is less than the center pixel of the above gray scaled image, therefore the CLBP_C is:

CLBP_C = 1

For CLBP_S:

| CLBP_S = | 1 | -1 | -1 |
| --- | --- | --- | --- |
|  | -1 |  | -1 |
|  | 1 | 1 | 1 |

Reading in clockwise direction:

CLBP_S = 00011101

For the CLBP_M:

The magnitude difference matrix is:

| CLBP_M*_difference_* = | 109 | 37 | 146 |
| --- | --- | --- | --- |
|  | 73 |  | 91 |
|  | 0 | 73 | 54 |

By taking the threshold of 72.87, we get

| CLBP_M = | 1 | 0 | 1 |
| --- | --- | --- | --- |
|  | 1 |  | 1 |
|  | 0 | 1 | 0 |

Reading in clockwise direction:

CLBP_M = 01101011

It is noteworthy that the process for RECM transformation into feature vector using CLBP is the same as PSSM transformation.

# **Text S4. Qualitative Characteristics for Amino Acid Residues**

**Table S4.** Qualitative Characteristics for amino acid residues based on three distinct groups.

| Physiochemical Attributes | Group 1 | Group 2 | Group 3 |
| --- | --- | --- | --- |
| Hydrophobicity | Polar | Neutral | Hydrophobicity |
|  | R,K,E,D,Q,N | G,A,S,T,P,H,Y | C,L,V,M,F,W |
| Normalized van der waals volume | 0-2.78 | 2.94-4.0 | 4.03-8.08 |
|  | G,A,S,T,P,D | N,V,E,C,Q,I,L | M,H,K,F,R,Y,W |
| Polarity | 4.9-6.2 | 8.0-9.2 | 10.4-13.0 |
|  | L,I,F,W,C,M,V,X | P,A,T,G,S | H,Q,R,K,N,E,D |
| Polarizability | 0-1.08 | 0.128-0.186 | 0.219-0.409 |
|  | G,A,S,D,T | C,P,N,E,Q,I,L | K,M,H,F,R,Y,W |
| Charge | Positive | Neutral | Negative |
|  | K,R | A,N,C,Q,GH,I,L,M,F,P,S,T,W,Y,V | D,E |
| Secondary structure | Helix | Strand | Coil |
|  | E,A,L,M,Q,KR,H | V,I,TC,W,F,T | G,N,P,S,D |
| Solvent-accessible | Buried | Exposed | Intermediate |
|  | A,L,FC,G,I,V,W | R,K,QE,N,D | M,S,PT,H,,Y |

# **Text S5. Binary Tree Growth Algorithm**

In this study, we used the Binary tree growth (BTG) algorithm to select the optimal feature set. The details of the BTG algorithm are as follows.

In the first step, the initial population of trees is arbitrarily generated and then the fitness value for each tree is calculated by using the following function:

$$Fitness=\beta*E_{r}+(1-\beta)*\frac{\left|S\right|}{\left|F\right|}$$

where $E_{r}$ is the learning error rate, $\beta$ is used to control both the prediction error and feature reduction and its value is between 0 and 1, $\left|S\right|$ represents the number of selected features, and $\left|F\right|$ denotes the total features in the dataset.

Next, the fitness values are used to sort out the population of trees in ascending order. The first tree group receives the best $T_{1}$ trees, and the following mathematical formula is used to generate the new tree in this group:

$$N_{i}^{t+1}=\frac{N_{i}^{t}}{\theta}+rN_{i}^{t}$$

where $N_{i}$ at order $i$ in the population denotes the tree (solution), $\theta$ denotes the trees diminution rate of power, $r$ is the randomly disturbed number between $[0,1]$ and $t$ is the number of current iteration. The current tree is replaced if the newly constructed tree has a better fitness score otherwise, it is stored for the next generation.

In the next step, $T_{2}$ trees are assigned to the second group, and for each tree, the two closest trees from the first and second groups are determined using the Euclidian distance:

$$d_{i}=\left(\sum_{i=1}^{T_{1}+T_{2}} (N_{T_{2}}^{t}-N_{i}^{t})^{2}\right)^{\frac{1}{2}}$$

where $N_{T_{2}}$ denotes the present tree and $N_{i}$ represents the tree at $i\text{-th}$ position in the population. It is worth mentioning that the distance becomes infinite when $N_{T_{2}}=N_{i}\text{ }$, $\text{where }T_{2}=i$. Then, the two nearest trees $x_{1}$,$x_{2}$ with minimum$d_{i}$ are selected and the following equation is used to compute the linear combination of the selected trees:

$$Q=\lambda x_{1}+(1-\lambda)x_{2}$$

where the parameter $\lambda$ is employed to control the impact of the closest tree. The location of the tree in the second group is updated using:

$$N_{T2}^{t+1}=N_{T2}^{t}+\alpha Q$$

where $\alpha$ is the angle distribution between [0,1]. The $T_{3}$ worst trees in the third group are eliminated and substituted with the new trees. Equation (10) can be used to compute the $T_{3}$:

$$T_{3}=T-T_{1}-T_{2}$$

where $T$ is the population size.

Employing a masked operator, $T_{4}$ new trees are constructed within the last group around the best trees. These newly constructed trees are then added to the population and the fitness values are used to sort the merged population in ascending order. In the subsequent iteration, the best $T$ trees are then selected to represent the new population. The process is reiterated until the termination criterion is met and finally, the universally finest tree is chosen as the best solution.

To select the optimal feature sets, the BTG algorithm utilizes a transfer function to translate the location of the trees into probability values ranging between 0 to 1. A large probability number means there will be a larger possibility that the feature will be selected. In the present study, we used the sigmoid function as the transfer function which can be expressed as:

$$\delta(q_{id}^{t})=\frac{1}{1+e^{-q_{id}^{t}}}$$

where $q$ denotes the $d\text{-th}$dimensionality of the search space. The location of the tree is updated depending on the value of the probability described below:

$$q_{id}^{t+1}=\left\{\begin{matrix} 1,\text{ if }\partial<\delta_{id}^{t} \\ 0,\text{ otherwise} \end{matrix}\right.$$

Where $\partial$ is a random number between 0 and 1. The procedure of the mask operation in the BTG algorithm is shown in Table S3.

| **Table S5**. A simple example showing the procedure mask operation. | | | | | | |
| --- | --- | --- | --- | --- | --- | --- |
| New tree | 1 | 1 | 0 | 0 | 0 | 0 |
| Mask Operator | 0 | 1 | 0 | 1 | 1 | 0 |
| Random finest tree | 0 | 1 | 0 | 1 | 0 | 1 |
| New Tree after masking | 1 | 1 | 0 | 1 | 0 | 0 |

In this study, we employed the k-nearest neighbor (KNN) machine learning algorithm in the process of fitness evaluation because it is simple yet efficient and faster. In KNN, the value k is empirically set to 5.

# **Text S6. Details of SnBiLSTM**

In order to address the shortcomings of traditional recurrent neural networks (RNNs), such as capturing and learning long-term dependencies, vanishing and exploding gradients during training, Long-Short-Term Memory (LSTM) networks have emerged as a novel RNN architecture [46]. The LSTM network layers are composed of memory blocks that are recurrently interconnected with memory cells. These memory cells incorporate multiple gate controllers which manage the flow of information. These gate controllers determine when to forget or retain the previously learned hidden information and when to update the cells. This approach allows the network to efficiently learn and utilize the temporal information.

Despite the LSTM networks' ability to capture and learn the long-term dependencies, they are limited by their unidirectional processing nature, typically from start to end. Therefore, the Bidirectional Long Short-Term Memory, simply known as BiLSTM, which is a variant built upon the strengths of LSTM, addresses the above-mentioned limitation by incorporating forward and backward LSTMs working together. The forward LSTM processes the sequence information from start to end while the backward LSTM operate on a reversed copy of sequence information, examining it from end to start. Consequently, BiLSTM leverages both past and future information along with the present information making it more effective. Both the forward and backward LSTMs function independently and the information extracted by both LSTMs layers are then coupled before inputting to the output layer. The forward, backward and output information can be mathematically represented as:

$$h_{t}^{forward}=\sigma(U_{h}^{forward}\cdot m_{t}+W_{h}^{forward}\cdot h_{t-1}^{forward}+b_{h}^{forward})$$

$$h_{t}^{back}=\sigma(U_{h}^{back}\cdot m_{t}+W_{h}^{back}\cdot h_{t-1}^{back}+b_{h}^{back})$$

$$y_{t}=\sigma(V_{h}^{forward}\cdot h_{t}^{forward}+V_{h}^{back}\cdot h_{t}^{back}+b_{y})$$

where $h_{t}^{forward}$ and $h_{t}^{back}$ denote the hidden state at time step $t$ in the forward and backward LSTMs; $U_{h}^{forward}$ and $U_{h}^{back}$ are the weight matrices associated with the input $m_{t}$; $W_{h}^{forward}$ and $W_{h}^{back}$ are the weight matrices associated with the previous hidden state $h_{t-1}^{forward}$ and $h_{t-1}^{back}$; respectively; $b_{h}^{forward}$ and $b_{h}^{back}$ represent the bias vector added to the weighted sum; $y_{t}$ denotes the output at time step $t$; $V_{h}^{forward}$ and $V_{h}^{back}$ are the weight matrices linked with the forward hidden state $h_{t}^{forward}$ and backward hidden state $h_{t}^{back}$, respectively; $b_{y}$ represent the bias vector added to the weighted sum and $\sigma$ is an activation function.

Training BiLSTM networks can be computationally expensive and time-consuming. Numerous risks are associated with training BiLSTM networks, including vanishing gradients and limitations in generalization to unseen samples. Additionally, searching the optimal configuration of hyperparameters through gradient descent can be challenging as well. one effective approach to cope with training time issues involve the normalization of neurons activities within the network. Therefore, in this work, batch normalization was implemented as a self-normalization approach to speed up the training process by incorporating additional normalization layers within the BiLSTM network [47]. This self-normalization technique can standardize each aggregated input by standard deviation and its mean across the training data. Table S4 provides all the hyperparameters for training the SnBiLSTM Model.

| **Table S6.** Hyper Parameters of the SnBiLSTM Model. | |
| --- | --- |
| **Hyper-parameters** | **Values** |
| No of units in LSTM | 128,64,32 |
| return_sequences | True |
| L2 Regularization | 0.0001 |
| Optimizer | Adam, SGD |
| Learning rate | 0.001 |
| Dense Layer unit | 1 |
| Batch size | 16, 32, 64 |
| Batch Norm | Yes |
| Epochs | 40,60 |
| Factor | 0.1 |
| Patience | 5 |
| Dropout rates | 0.25 - 0.35 |
| Loss function | Binary cross entropy |
| Activation function | Tanh, Sigmoid |

# **Text S7. Mathematical Notations for Performance measures**

Following are the mathematical notations for the Accuracy (*Acc*), Sensitivity (*Sen*), Specificity (*Spe*) and the Matthew correlation coefficient (*MCC*).

$$Acc=\frac{TP+TN}{TP+FP+TN+FN}$$

$$Sen=\frac{TP}{TP+FN}$$

$$Spe=\frac{TN}{TN+FP}$$

$$MCC=\frac{TP\cdot TN-FP\cdot FN}{\sqrt{(TP+FP)\cdot(TP+FN)\cdot(TN+FP)\cdot(TN+FN)}}$$

where $TP$ represents true AVPs that are correctly predicted by the model as positive instances, $TN$ represents true non-AVPs that are correctly classified by the model as negative instances, $FN$ represents AVPs that are mistakenly predicted as non-AVPs and $FP$ represents non-AVPs that are mistakenly predicted as AVPs.

# **Text S8. Model Visualization**

To further examine the discriminative power of the extracted features, we employed Uniform Manifold Approximation and Projection (UMAP) based data approach to visualize the separation between targeted classes. The Visualization results are illustrated in Figure S3 (A-F). The UMAP projects the high-dimensional feature space into a two-dimensional space. This visualization is significant in revealing the effectiveness of each feature encoding method employed in developing an efficient prediction model.


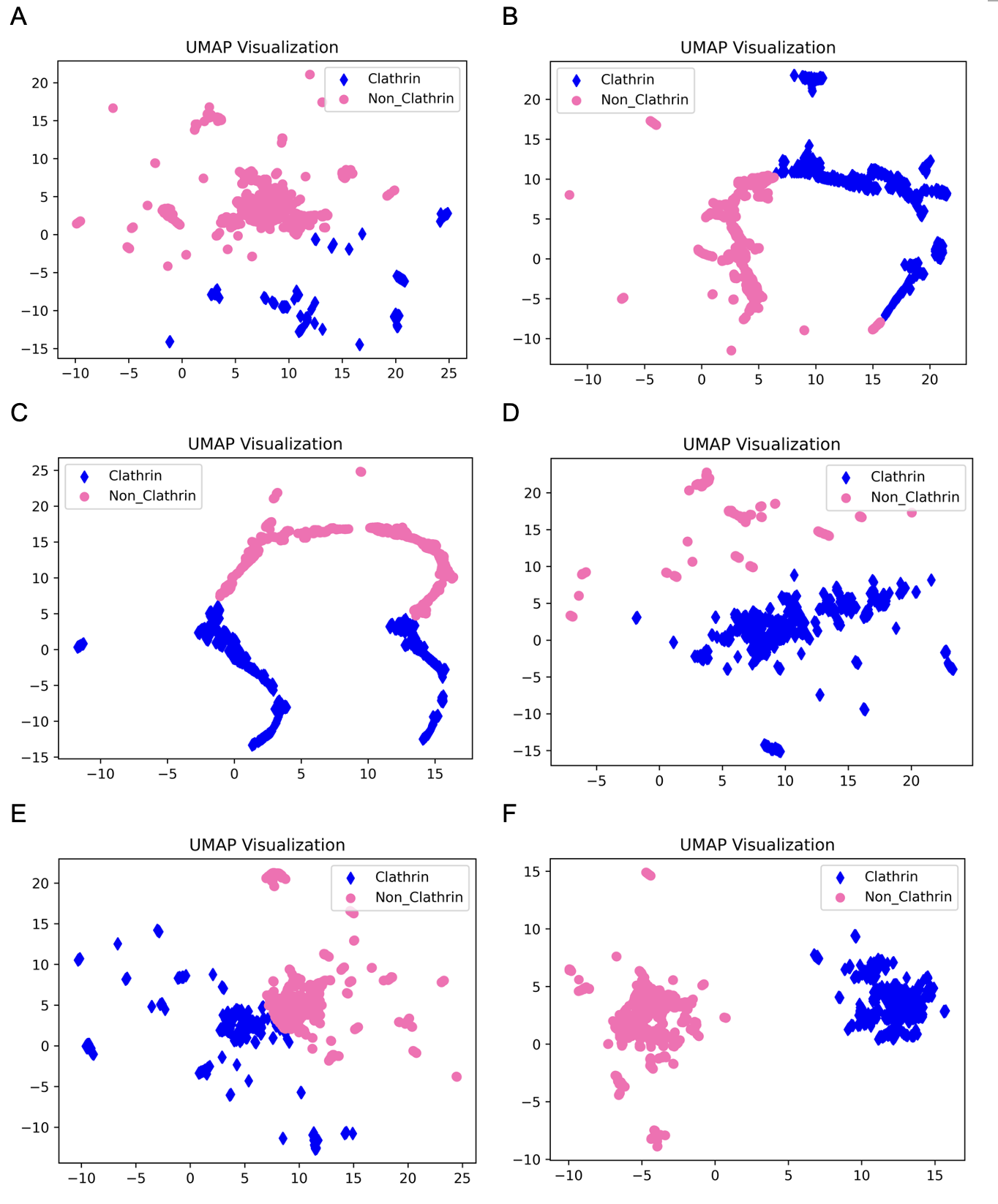


**Figure S3:** UMAP visualization of extracted features A) ESM, B) PSSM-CLBP, C) RECM-CLBP, (D) QLC, (E) W-Features (F) W-Features+BTG

After analyzing the individual and hybrid features in Figure S3(A-E), some overlap among different samples is observed, implying that some enhancements are essential to clearly discriminate the Clathrin (blue diamond) and non-Clathrin (pink circles) protein classes. After employing the BTG feature selection, as shown in Figure S3(F), it can easily be observed that that the separation between the targeted classes is strong, highlighting the effectiveness of the feature selection process and the proposed method. This visualization analysis also provides additional evidence of the computational and biological relevance of the features for the clathrin prediction task.

# **Text S9. Model Interpretation**

To identify the contributions of individual features in the optimal set obtained by BTG, we employed SHapley Additive exPlanations (SHAP) analysis. SHAP highlights the global importance of the features based on their contributions. Feature contributions are examined using SHAP values, derived from the aggregated Shapley values in game theory, to provide global insights. Figure S4 provides summary of the most contributory features identified via SHAP analysis. The features such as ESM-93, PSSM-CLBP-90, and PSSM-CLBP-199 showed the highest contributions to the prediction performance of the model. These features capture critical sequence-level information, evolutionary and spatial patterns. The features, derived from the RECM-CLBP such as RECM-CLBP-46, captured structural information through, while the qualitative characteristics features, such as QLC-15 encoded physiochemical properties essential to clathrin proteins. The positive SHAP values (high values), which are at the right side of the vertical line, push the prediction toward the positive class i.e. Clathrin proteins. The negative values (low values), the left side of the vertical line, push the prediction toward the negative class i.e. non-Clathrin proteins. These SHAP values highlights how high (red color) and low (blue color) feature values affect the predictions, further indicating that biologically relevant properties such as evolutionary information, secondary structure and hydrophobicity, significantly contribute to discriminating clathrin and non-clathrin proteins. This SHAP analysis validates the feature selection procedure by emphasizing the biological relevance of top-ranking features while improving the interpretability of the TargetCLP.


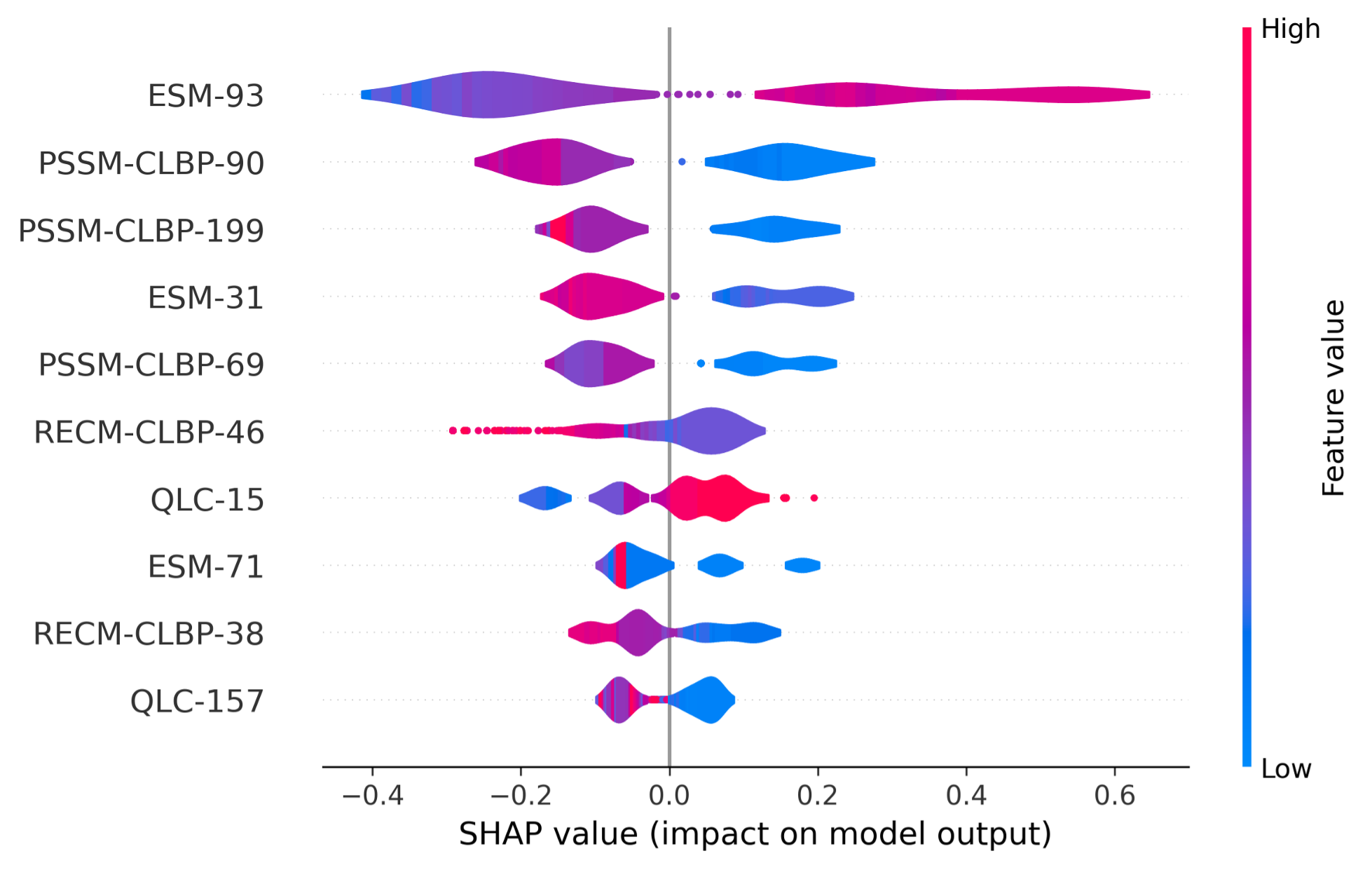


**Figure S4:** SHAP analysis based on highly informative features

# **Text S10. Comparative Results on the Independent Dataset**

| **Table S7.** Performance comparison of TargetCLP with the past work on the independent dataset CL485. | | | | | |
| --- | --- | --- | --- | --- | --- |
| **Predictor** | ***Acc* (%)** | ***Sen* (%)** | ***Spe* (%)** | ***MCC*** | **AUC** |
| Le et al. predictor | 91.80 | 92.20 | 91.20 | 0.83 | - |
| TargetCLP | 92.78 | 89.42 | 95.72 | 0.85 | 0.96 |

We can observe in Table S5 that our Proposed TargetCLP has better generalization ability than the existing method proposed by Le et al. This once again proves that our proposed method is competitive and useful for forecasting of clathrin proteins.

# **Text S11. Computational Efficiency and Scalability**

As mentioned in the manuscript that the key goal of the current study is on the developing and evaluating the predictive performance of the proposed TargetCLP as the field of the computational predictors for Clathrin proteins is still emerging and there is need for improving the predictive accuracy and interpretability. However, here we underline the general resource requirements needed during our experiments and the scalability of the methodology.

In our experiments. The TargetCLP model training benefits from Intel(R) Core(TM) i9-13905 @ 2.6GHz with 32GB of RAM and GPU of 8GB NVIDIA GeForec RTX 4060. We used MATLAB R2019b, Spyder 5 and Jupiter Notebook. Similarly, the modular pipeline design of the TargetCLP enables for batch processing and parallel computation, slowing scalability.

As mentioned in the “Conclusion” section of the manuscript, future work will emphasis on optimizing the computational complexity and runtime of the TargetCLP to facilitate its applicability to large-scale protein datasets.
